# The Many Fronts of Battling Obstructive Sleep Apnea

**DOI:** 10.7759/cureus.16937

**Published:** 2021-08-06

**Authors:** Waiz Wasey, Neha Wasey, Sharefi Saleh, Imad Aziz

**Affiliations:** 1 Family and Community Medicine, Southern Illinois University School of Medicine, Springfield, USA; 2 General Practice, Shadan Institute of Medical Sciences, Hyderabad, IND; 3 Family Medicine, Ruth Temple Health Center, Los Angeles, USA; 4 Family Medicine, Mercyhealth, Beloit, USA

**Keywords:** risk factors for obstructive sleep apnea (osa), worsening osa, anterior cervical discectomy fusion, obesity, continuous positive airway pressure (cpap), bipap, opioids

## Abstract

Obstructive sleep apnea (OSA) is a breathing disorder during sleep secondary to collapsing upper airways that leads to a significant decrease or a complete cessation of airflow despite an effort to breathe. As the name suggests, an obstruction, likely caused by an inherited narrow airway, is the most common cause. But there are less known factors that may contribute to the worsening of OSA. We present a case of very severe OSA contributed by weight gain, opioid use, and anterior cervical discectomy and fusion (ACDF), in addition to a genetically narrow airway. This case highlights the importance of battling OSA on many different fronts. Our patient eventually was able to stop positive airway pressure (PAP) therapy, once the contributing factors were addressed appropriately.

## Introduction

Obstructive sleep apnea (OSA) is a common yet underdiagnosed sleep breathing disorder. In the general population, its prevalence is estimated to be 34% in men and 17% in women [1]. It is the decrease or complete cessation of airflow despite efforts to breathe. OSA is influenced by various risk factors, which may or may not be modifiable. These include male sex, advanced age, race, obesity, use of certain medications, endocrine disorders, nasal obstruction, and surgical procedures [2]. OSA is commonly treated with positive airway pressure (PAP) therapy. We present a case of a 45-year-old female who was on bilevel PAP for very severe OSA with multiple modifiable risk factors. Once these factors were addressed appropriately, she was able to come off of PAP therapy.

## Case presentation

A 45-year-old female with a past medical history of complex regional pain syndrome (CRPS), gastroesophageal reflux disease (GERD), depression, anxiety, and a history of severe OSA was seen in our sleep clinic for persistent daytime somnolence and worsening symptoms of OSA despite using bilevel PAP. Her relevant past medical history included the anterior cervical discectomy and fusion (ACDF) procedure done in November 2017.

She was diagnosed with OSA following polysomnography (PSG) in 2019. This was a split night study where the second half of the night involved PAP titration to find optimum pressure to control the OSA. The results from this diagnostic test were as follows (Table 1):

**Table 1:** Initial polysomnography done in July 2019. PAP: Positive airway pressure.

Sleep parameters	
Total sleep time	490 mins
Apnea Hypopnea Index (AHI)	52/hr
Lowest oxygen saturation	77%
Periodic limb movements	0.4/hr
PAP titration result	Bilevel PAP 17/11 cmH2O

**Figure 1:**
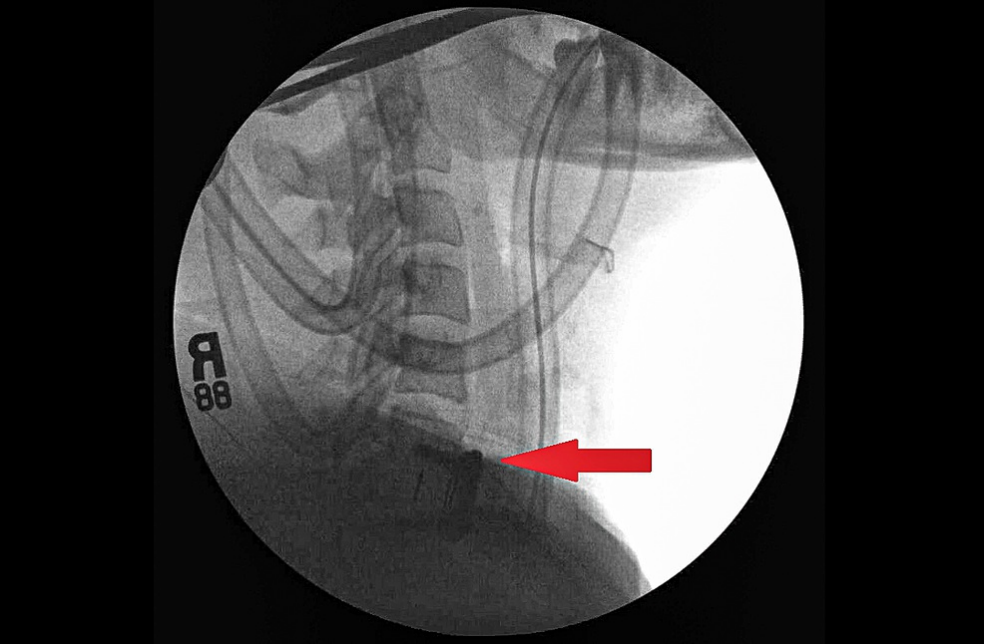
X-ray with fluoro imaging of anterior cervical fusion at C6-C7.

Prior to 2017, the patient denied any symptoms of snoring, daytime somnolence, or nonrestorative sleep. It was in November of 2017 when she had ACDF (Figure 1) done for chronic cervicalgia. Following the PSG in 2019, she was started on bilevel PAP at the settings of 17/11 cmH2O.

At the time of her initial evaluation with us, her weight was 238 lbs with a BMI of 39 kg/m2. She had a narrow airway with a Mallampati score of 4. She was also on clonazepam 0.5 mg twice daily for anxiety, Dilaudid 4 mg every six hours, morphine ER 15 mg twice daily and morphine ER 30 mg twice daily for chronic pain. She had complained of bloating and swallowing air (aerophagia) from using the bilevel PAP and, despite therapy, had persistent daytime symptoms of sleepiness, with an Epworth score of 19.

On downloading the compliance data, the following parameters were noted (Table 2):

**Table 2:** Downloaded compliance report from PAP machine. AHI: Apnea Hypopnea Index; PAP: Positive airway pressure.

Compliance report from bilevel PAP	
Days used	12/30 days
Residual AHI	11.7/hr
Central apnea index	10.8/hr

The download report indicated that the patient was developing treatment-emergent central apnea on top of her OSA. This was most likely due to the use of opioids. Pressure changes were done to decrease the inspiratory PAP pressures to avoid further aerophagia and reduce events of central apnea. Her settings were brought down to 15/11 cmH2O.

After a month of follow-up, she still complained of persistent daytime sleepiness and struggled to keep her bilevel PAP on throughout the night. Due to her CRPS, it was difficult for her to get a good night’s rest due to pain and discomfort from the PAP mask. Since she was on higher pressure, a full face mask had to be used. This also made her feel claustrophobic, secondary to her anxiety.

Since there were modifiable factors involved in worsening the patient's OSA, we took steps to address them along with treating her sleep apnea. For the next year, the patient worked with specialists to wean herself off of opioids for pain control. She was now using buprenorphine buccal film to manage her CRPS. She also worked with weight loss specialists to lose weight and had decreased her BMI from 39 kg/m2 in September 2019 when she came to our clinic first, to 31 kg/m2 in April 2021.

At this point, we repeated a sleep study on the patient as she significantly lost weight to evaluate if her PAP pressure requirements would decrease. The thought was that lower pressures would help us use less invasive masks and provide the patient more comfort. The results of the repeat sleep study were as follows (Table 3). These are compared with her previous PSG results.

**Table 3:** Comparing old PSG (2019) with new PSG (2021). OSA: Obstructive sleep apnea; PSG: Polysomnography.

Polysomnography	July 2019	April 2021
Total sleep time	490 mins	514 mins
Apnea Hypopnea Index	52/hr	5.4/hr
Central apnea index	0/hr	0/hr
Lowest oxygen saturation	77%	93%
Interpretation	Severe OSA	Mild OSA

**Figure 2:**
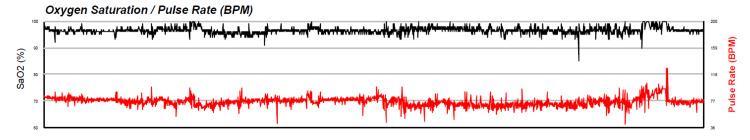
Oximeter tracing from the new sleep study (2021).

Her oxygen saturation graph from the recent sleep study was as follows (Figure 2), showing minimum desaturations:

Following the results of this study, the patient was taken off from PAP therapy with continued efforts to avoid opioid use and continue to lose weight. On a subsequent follow-up visit two months later, her Epworth score improved to 9. She was able to fall asleep easier with no claustrophobia or discomfort.

## Discussion

OSA affects 15-50% of the general population and increases the risk of cardiovascular and metabolic disease [1]. The collapse of the upper airway leads to a decrease or complete cessation of airflow during sleep. The most common cause for OSA is narrow upper airways. These are generally due to inherited bony structures such as craniofacial deformities, micro or retrognathia, and inferior positioning of the hyoid bone. In addition to these, there are modifiable factors that worsen OSA. These include male sex, advanced age, race, obesity, use of certain medications, endocrine disorders, nasal obstruction, and surgical procedures [2].

Head and neck surgeries, such as ACDF, have been known to precipitate OSA [3]. What is still unknown is if the OSA is temporarily secondary to the tissue swelling and weak pharyngeal plexus as a result of the surgery or a permanent complication of the procedure. In our case, the symptoms of OSA came on after ACDF was done. It was likely the precipitating and aggravating factor. The narrow airway from the plate placement and the extra fat around the patient's neck due to significant weight gain made her OSA worse. It has been time and again established that weight has a bidirectional relationship with OSA. About 58% of moderate-to-severe OSA is due to obesity [4]. Since obesity is a modifiable risk factor, weight loss can help reduce the severity of OSA but not cure it [5]. In our case, the significant weight loss from a BMI of 39 to 31, definitely played a significant role in reducing the severity of OSA.

The use of opioids has soared significantly since the 1990s. These work by interacting with the mu, delta, or kappa receptors in the brain [6]. Since these cause CNS depression, it can lead to respiratory depression as well. It has been well established that regular opioid use interferes with sleep architecture as well as worsening sleep-disordered breathing [7]. Although opioids can worsen OSA, they mainly cause central sleep apnea (CSA). CSA is when there is a brief disconnect between the respiratory centers in the brain and the lungs, leading to no breathing effort and hence apnea. As evident in our patient’s compliance report, she developed CSA which did not exist on her original PSG report. This was most likely due to the respiratory depressing effect of the medicines. Once she stopped using opioids for pain control, her repeat PSG did not show any significant CSA and her OSA improved significantly as well.

In the majority of cases, OSA is not curable. Even in our case, the OSA still persisted after addressing the risk factors, but the severity decreased significantly. The gold standard treatment for moderate or severe OSA is PAP therapy [8]. The PAP helps maintain patent airways so that patients with collapsing airways can breathe without difficulty. However, there are numerous options to treat mild OSA. Most of these are less cumbersome. PAP therapy comes with difficulties of usage. In our patient, she struggled with pain from mask discomfort and aerophagia. Other complications include face acne, mask leaks causing dry eyes, chest pain, bloating, infections from improper cleaning and not to mention the cost of maintaining the PAP device. For mild OSA, other treatment options include mandibular advancement device [9], positional therapy [10], and the novel eXciteOSA treatment. These also have better compliance than PAP machines. Our patient found a significant improvement in her quality of sleep from stopping PAP therapy. Since her Apnea Hypopnea Index (AHI) was 5.4/hr, we did not feel the need to continue PAP therapy. We encouraged her to avoid opioids and continue weight loss.

Epworth sleepiness scale is a subjective screening tool to measure the daytime impairment from inadequate sleep [11]. It consists of subjectively measuring chances of dosing off in eight different scenarios and is scored from 0 to 24. A score of greater than 10 is significant for daytime impairment as a result of underlying sleep disorder (Table 4).

**Table 4:** Epworth sleepiness score screening tool.

Chances of dozing off during	No chance	Mild chance	Moderate chance	High chance
Sitting and reading	0	1	2	3
Watching TV	0	1	2	3
Sitting inactive in a public place	0	1	2	3
Being a passenger in a motor vehicle for an hour or more	0	1	2	3
Lying down in the afternoon	0	1	2	3
Sitting and talking to someone	0	1	2	3
Sitting quietly after lunch	0	1	2	3
Stopped for a few minutes in traffic while driving	0	1	2	3
Total				

Our patient initially had a score of 19. This improved to 9 on her latest visit. This indicates that addressing the risk factors not only helped reduce her OSA, but also improve her daytime functioning.

## Conclusions

OSA is a breathing condition mostly caused by narrow airways but may be worsened by multiple modifiable risk factors. Once a patient is diagnosed with OSA and started on treatment with PAP therapy, the modifiable risk factors should not be ignored. Continued efforts should be made in addressing the risk factors alongside PAP treatment. If our patient had not addressed her weight or opioid use, her OSA would have remained severe and she would have struggled with using PAP. The purpose of this case report is to demonstrate that OSA needs to be battled on many fronts, and the job is not done by starting PAP therapy alone.
